# Cruciferous Vegetables Intake Is Associated with Lower Risk of Renal Cell Carcinoma: Evidence from a Meta-Analysis of Observational Studies

**DOI:** 10.1371/journal.pone.0075732

**Published:** 2013-10-28

**Authors:** Jun Zhao, Long Zhao

**Affiliations:** 1 Department of Nephrology, Shandong Weifang People's Hospital, Weifang, China; 2 Department of Nephrology, The Second Hospital of Shandong University, Jinan, China; Deutsches Krebsforschungszentrum, Germany

## Abstract

**Background:**

Epidemiologic studies have evaluated the association between cruciferous vegetables(CV) intake and the risk of renal cell carcinoma(RCC); however, the existing results are controversial. The aim of this meta-analysis was to investigate the association between CV intake and RCC risk.

**Methods:**

A literature search was carried out using PUBMED and EMBASE database between January 1966 and March 2013. Fixed-effect and random-effect models were used to estimate summary relative risks (RR) and the corresponding 95% confidence intervals (CIs). Potential sources of heterogeneity were detected by meta-regression. Subgroup analyses, sensitivity analysis and cumulative meta-analysis were also performed.

**Results:**

A total of 12 studies (six cohorts, six case–control) contributed to the analysis, involving 1,228,518 participants and 5,773 RCC cases. When all studies were pooled, we observed a significantly inverse association between CV intake and RCC risk (RR = 0.81, 95% CI [0.72, 0.91]). This association was also significant when analyses were restricted to six high-quality studies (RR = 0.89, 95% CI [0.82, 0.98]). In subgroup analyses, CV intake was significantly associated with reduced RCC risk among studies conducted in America (RR = 0.77, 95%CI [0.70, 0.86]); however, CV intake had no significant association with RCC risk among studies conducted in Europe (RR = 0.87, 95%CI [0.71, 1.07]). Furthermore, sensitivity analysis confirmed the stability of results.

**Conclusions:**

The findings of this meta-analysis suggested that high intake of CV was inversely associated with RCC risk among Americans. More studies, especially high quality cohort studies with larger sample size, well controlled confounding factors are warranted to confirm this association.

## Introduction

Kidney cancer among adults consists of malignant tumors arising from the renal parenchyma and renal pelvis. Renal cell carcinoma(RCC) accounts for about 90% of adult kidney cancer and 3% of adult malignancies [1]. The age-adjusted incidence rate of the kidney cancer was 15.1 per 100,000 men and women per year, and the age-adjusted death rate was 4.0 per 100,000 men and women per year [2]. The incidence of RCC has been steadily increasing in the United States, doubling over the past three decades [3]. Although cigarette smoking, obesity, and hypertension are established risk factors, the etiology of RCC is largely unknown [1], [4]. A recent pooled analysis of 13 prospective studies reported that fruit and vegetable consumption was associated with a reduced risk of RCC, and carotenoids present in fruit and vegetable may partly contribute to this protection [5].

Cruciferous vegetables (CV) are a group of vegetables named for their cross-shaped flowers, including cabbage, broccoli, cauliflower, brussels sprouts and other members of the family. CVs are unique in that they are rich sources of sulfur-containing compounds known as glucosinolates (GLS) [6], [7], [8], [9]. Chopping or chewing cruciferous vegetables results in the formation of bioactive GLS hydrolysis products, such as isothiocyanates (ITCs) and indole-3-carbinol(I3C), which may contribute to reduce risk of RCC. Nonetheless, the anti-carcinogenic actions of GLS are commonly attributed to ITCs. ITCs can compound sulforaphane, which may help prevent cancer by enhancing the elimination of potential carcinogens from the body and increasing the transcription of tumor suppressor proteins, including those silenced by epigenetic mechanisms [9], [10], [11], [12], [13], [14], [15]. Nevertheless, the epidemiological results of whether intake of CV may protect against RCC are still controversial, with some epidemiological studies having not identified significant effect [16], [17], [18], [19], [20], [21], [22], [23], [24], others having described an significant reduced RCC risk [25], [26], [27]. To our knowledge, a comprehensive and quantitative assessment of the association between CV intake and RCC risk has not been reported. Therefore, we carried out a meta-analysis on all cohort and case-control studies to evaluate the relationship between CV intake and RCC risk.

## Materials and Methods

### Data sources and searches

The meta-analysis was undertaken in accordance with the Preferred Reporting Items for Systematic Reviews and Meta-Analyses (PRISMA) (Checklist S1. PRISMA Checklist) [28]. A literature search was carried out using PUBMED and EMBASE database between January 1966 and March 2013. There were no restriction of origin and languages. Search terms included: “brassicaceae” or “brassica” or “cruciferous vegetables” or “broccoli” or “cabbage” or “cauliflower” or “brussels sprouts” or “mustard plants” or “sauerkraut” or “cole slaw” or “collards” or “bok choy” or “turnip greens” or “vegetables” and “renal” or “kidney” and “cancer” or “neoplasm” or “malignancy”. The reference list of each comparative study and previous reviews were manually examined to find additional relevant studies.

### Study selection

Two reviewers independently selected eligible trials. Disagreement between the two reviewers was settled by discussing with the third reviewer. Inclusion criteria were: (i) used a case-control or cohort study design; (ii) evaluated the association between CV intake and RCC risk. When there were multiple publications from the same population, only data from the most recent report were included in the meta-analysis and remaining were excluded. Studies reporting different measures of RR like risk ratio, rate ratio, hazard ratio (HR), and odds ratio (OR) were included in the meta-analysis. In practice, these measures of effect yield a similar estimate of RR, since the absolute risk of RCC is low.

### Data extraction

The following data was collected by two reviewers independently using a purpose-designed form: name of first author, publishing time, country of the population studied, study design, study sample size, adjusted effect estimates for highest versus lowest level of intake, confounding factors for matching or adjustments.

### Methodological quality assessment

We used Newcastle-Ottawa scale to assess the methodologic quality of cohort and case-control studies. The Newcastle-Ottawa Scale contains eight items that are categorized three categories: selection (four items, one star each), comparability (one item, up to two stars), and exposure/outcome (three items, one star each). A “star” presents a “high-quality” choice of individual study. With consideration that there is a correlation between caloric intake and nutrient consumption, and possibly a direct or indirect causal relation between caloric intake and RCC risk, the scoring system was modified by adding an item in which a study with data analysis that used an energy-adjusted residual or nutrient-density model received an additional star [29]. Hence, the full score was 10 stars, and the high-quality study was defined as a study with≥7 awarded stars.

### Data synthesis and analysis

Heterogeneity was assessed using the Cochran Q and I^2^ statistics. For the Q statistic, a P value<0.10 was considered statistically significant for heterogeneity; for the I^2^ statistic, heterogeneity was interpreted as absent (I^2^: 0%–25%), low (I^2^: 25.1%–50%), moderate (I^2^: 50.1%–75%), or high (I^2^: 75.1%–100%) [30]. Some studies presented individual risk estimates according to the different types of CV and did not report the effect of total CV intake. In this situation, the study-specific effect size in overall analysis was calculated by pooling the risk estimates of the various CV types, using the inverse-variance method [31]. For studies that reported results separately for males and females, but not combined, we pooled the results using a fixed-effect model to obtain an overall combined estimate before combining with the rest of the studies [32]. Subgroup analyses were carried out according to (i) study design ( cohort versus case-control studies), (ii)geographic location (Europe versus America), (iii) gender (male verus female), (iiii) number of adjustment factors (n≥8 versus n≤7), adjustments for smoking status(yes, no), adjustment for alcohol intake (yes, no), adjustment for meat intake (yes, no), adjustment for total energy intake (yes, no) , adjustment for hypertension status (yes, no). Pooled RR estimates and corresponding 95% CIs were calculated using the inverse variance method. When substantial heterogeneity was detected(I^2^≥50%), the summary estimate based on the random-effect model (DerSimonian-Laird method) [33] was reported, which assumes that the studies included in the meta-analysis had varying effect sizes. Otherwise, the summary estimate based on the fixed-effect model (the inverse variance method) [34] was reported, which assumes that the studies included in the meta-analysis had the same effect size. We carried out sensitivity analyses by excluding one study at a time to explore whether the results were strongly influenced by a specific study. To better investigate the possible sources of between-study heterogeneity, a meta-regression analysis was performed [35]. A univariate model was established, and then variables with P values≥0.1 were entered into a multivariable model. Cumulative meta-analysis was also performed to identify the change in trend of reporting risk over time. In cumulative meta-analysis, studies were chronologically ordered by publication year, then the pooled RRs were obtained at the end of each year. Publication bias was assessed using Begg and Mazumdar adjusted rank correlation test and the Egger regression asymmetry test [36], [37]. All analyses were performed using Stata version 11.0 (StataCorp, College Station, TX).

## Results

### Search results and characteristics of studies included in the meta-analysis

**Figure 1:**
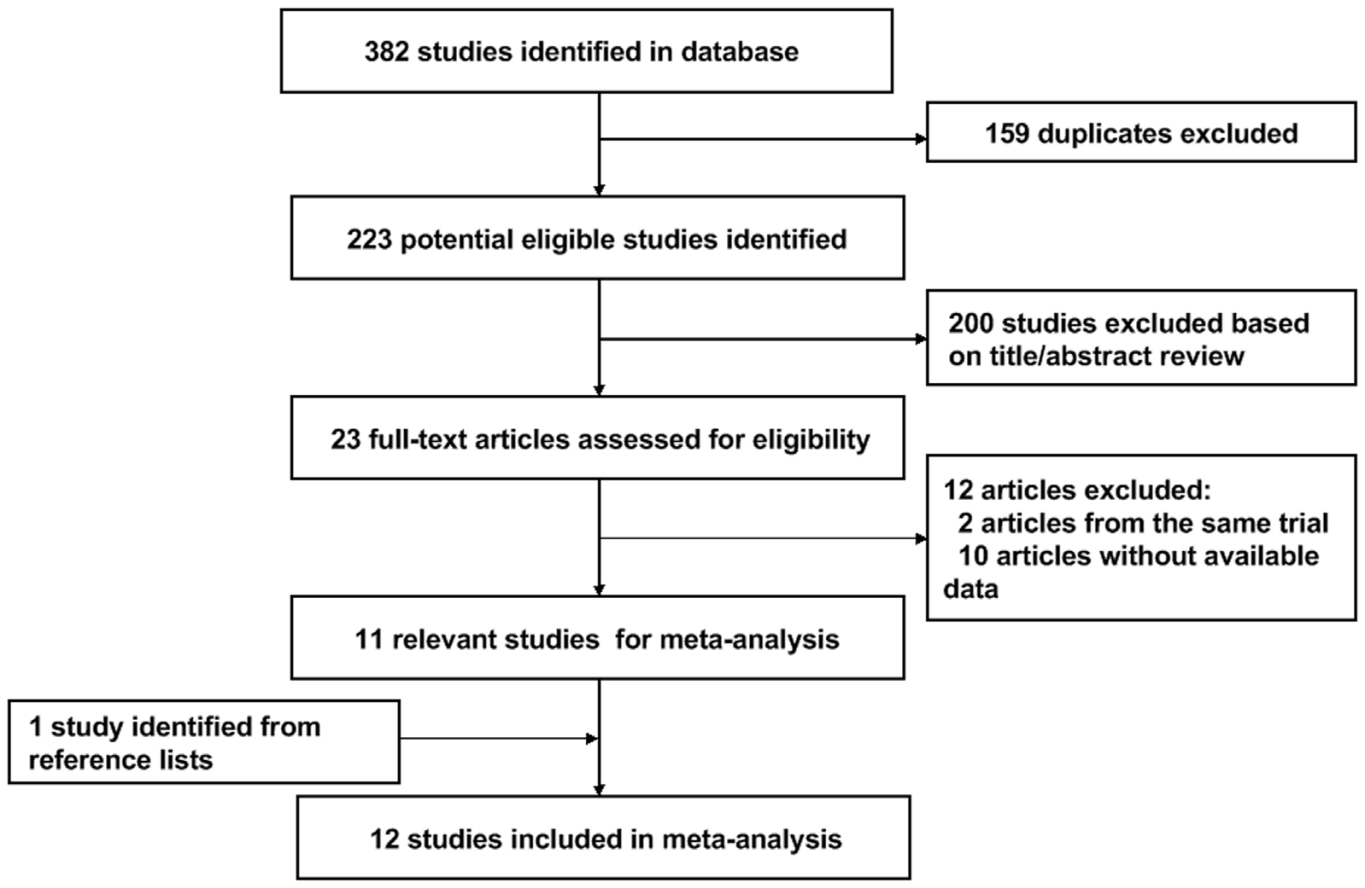
Flow diagram of screened, excluded, and analysed publications.

**Fig. 1** shows the flow diagram for study selection. A total of 382 citations were identified during the initial search. On the basis of the titles and abstracts, we identified 23 potentially relevant papers. After rigid evaluation, 12 studies were excluded (the reasons were described in **Fig. 1**). One additional study was identified from reference lists. At last, 12 studies published between 1990 and 2013 were included in the meta-analysis [16], [17], [18], [19], [20], [21], [22], [23], [24], [25], [26], [27], including six cohort studies, five population-based case–control studies, and one hospital-based case-control study. A total of 1,228,518 participants and 5,773 RCC cases were involved in the present meta-analysis. Baseline characteristics of the 12 eligible studies are shown in Table 1. Of them, six studies were conducted in America, and remaining six were in Europe. Three studies reported results separately for males and females [18], [19], [24], two studies investigated CV intake and RCC risk only in male or female population [16], [22]. Almost all of the risk estimates were adjusted for smoking status and body mass index(BMI), about half of the risk estimates were adjusted for hypertension, alcohol, and total energy intake (shown in Table 1). Table 2 and Table 3 summarizes the quality scores of cohort studies and case-control studies, respectively. The Newcastle-Ottawa Scale scores for the included studies ranged from 5 to 10, with a median 6.5.The median scores of cohort studies and case-control studies were 7.5 and 6, respectively. 6 studies (50%) were deemed to be of a high quality (≥7).

**Table 1:** Study characteristics of published cohort and case-control studies on cruciferous vegetables intake and risk of renal cell carcinoma.

Author	Year	Country	Study design	Cases/Subjects	Age(y)	Follow-up(y)	Confounders for adjustment
Daniel CR	2013	USA	cohort study	327/491,841	50–71	9(mean)	age, sex, education, race, marital status, family history of any cancer, BMI, smoking status, hypertension, diabetes, and intake of alcohol, red meat, and total energy; fruit,legumes, and whole grains
Brock KE	2012	USA	population-based case–control study	89/2,150	40–85	—	age, sex, proxy status, smoking status, BMI, blood pressure, alcohol consumption, fat consumption and energy
Bertoia M	2010	Finland	cohort study	69/27,062	50–69	19	age, smoking status, blood pressure, alcohol consumption and BMI
Hsu CC	2007	Eastern and Central Europe	hospital-based case-control study	1,065/2,574	20–79	—	age, country, gender, smoking status, education, BMI, hypertension medication use, alcohol consumption,and tertiles of total red meat and total white meat consumption
Lee JE	2006	USA	cohort study	248/136,587	30–55	14	BMI, history of hypertension, parity, history of diabetes, smoking status, multivitamin use, alcohol intake,and total energy intake
Weikert S	2006	Europe	cohort study	306/375,851	25–70	6.2(mean)	age, center, BMI, energy from fat sources, energy from non-fat sources,education, smoking, alcohol drinking and non-consumer status
Rashidkhani B	2005	Sweden	cohort study	122/61,000	40–76	13.4	age, BMI
van Dijk BA	2005	Netherlands	cohort study	275/120,852	55–69	9.3	age, sex, smoking status, BMI, history of hypertension and fruit consumption
Hu J	2003	Canada	population-based case–control study	1,279/6,449	≥20	—	age, province, education, BMI, alcohol use,smoking and total energy intake
Yuan JM	1998	USA	population-based case–control study	1,204/2,408	25–74	—	sex, date of birth, ethnicity, level of education, BMI, history of hypertension, smoking status, total grams of analgesics consumed over lifetime and use of amphetamines
Lindblad P	1997	Sweden	population-based case–control study	379/729	20–79	—	age, sex, BMI, cigarette smoking, and educational level.
Maclure M	1990	USA	population-based case–control study	410/1,015	≥30	7	age, sex, education, income, religious background, quetelet index, hypertention, heart disease, kidney stone, kidney infection

**Table 2:** Methodologic quality of cohort studies included in the meta-analysis.

Study and year	Selection				Comparability		Outcome				
	Representativeness of the exposed cohort	Selection of the unexposed cohort	Ascertainment of exposure	Demonstration that outcome of interest was not present at start of study	Study controls for age/gender	Study controls for additional factors	Assessment of outcome	Was follow-up long enough for outcomes to occur^1^	Adequacy of follow up of cohorts^2^	Data analysis that used an energy-adjusted residual or nutrient-density model	Total quality scores
Daniel CR 2013	☆	☆	☆	☆	☆	☆	☆	☆	☆	☆	10
Bertoia M 2010	-	☆	☆	☆	-	☆	☆	-	☆	-	6
Lee JE 2006	-	☆	☆	☆	-	☆	☆	☆	☆	☆	8
Weikert S 2006	-	☆	☆	☆	-	☆	☆	-	☆	☆	7
Rashidkhani B 2005	☆	☆	☆	☆	-	-	☆	☆	☆	-	7
van Dijk BA 2005	☆	☆	☆	☆	☆	☆	☆	☆	☆	-	9

1 A cohort study with a follow-up time >8 y was assigned one star.

2 A cohort study with a follow-up rate >75% was assigned one star.

**Table 3:** Methodological quality of case-control studies included in the meta-analysis.

Study and year	Selection				Comparability		Exposure				
	Adequate definition of cases	Representativeness of cases	Selection of control subjects	Definition of control subjects	Study controls for age/gender	Study controls for additional factors	Exposure assessment	Same method of ascertainment for cases and controls	Non-Response rate^1^	Data analysis that used an energy-adjusted residual or nutrient-density model	Total quality scores
Brock KE 2012	☆	☆	☆	☆	☆	☆	-	☆	-	☆	8
Hsu CC 2007	☆	-	-	☆	☆	☆	-	☆	☆	-	6
Hu J 2003	☆	-	☆	☆	-	☆	-	☆	-	☆	6
Yuan JM 1998	☆	-	☆	☆	-	☆	-	☆	-	-	5
Lindblad P 1997	☆	-	☆	☆	☆	☆	-	☆	-	-	6
Maclure M 1990	☆	-	☆	☆	☆	☆	-	☆	-	-	6

1 One star was assigned if there was no significant difference in the response rate between control subjects and cases by using the chi-square test (P>0.05).

### Main analysis

**Figure 2:**
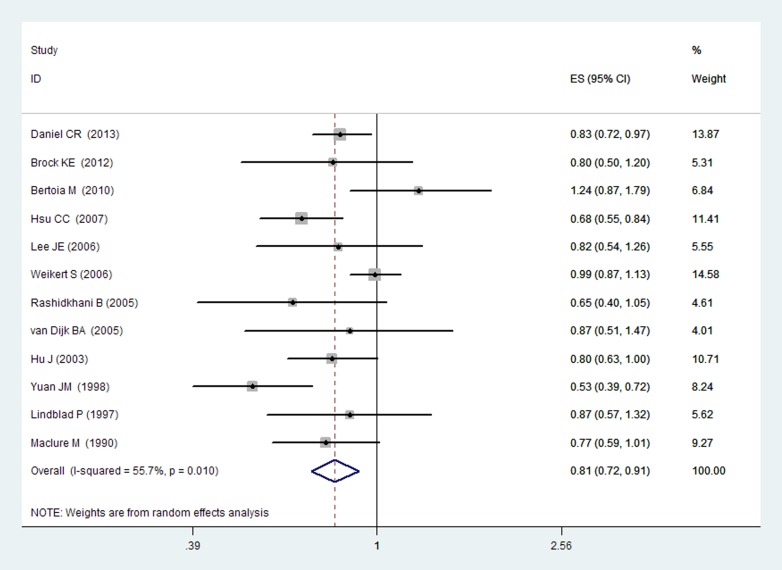
Forest plot: overall meta-analysis of CV intake and RCC risk. Squares indicated study-specific risk estimates (size of square reflects the study-statistical weight, i.e. inverse of variance); horizontal lines indicate 95% confidence intervals; diamond indicates summary relative risk estimate with its corresponding 95% confidence interval.

Since significant heterogeneity (I^2^ = 55.7%, q = 0.01) was observed, the random-effects model was chosen over a fixed-effects model and we found that high CV intake (comparing the highest with the lowest category) was associated with a reduced RCC risk (RR = 0.81, 95% CI [0.72, 0.91], Fig. 2).

### Subgroup analyses, sensitivity analysis and cumulative meta-analysis

**Figure 3:**
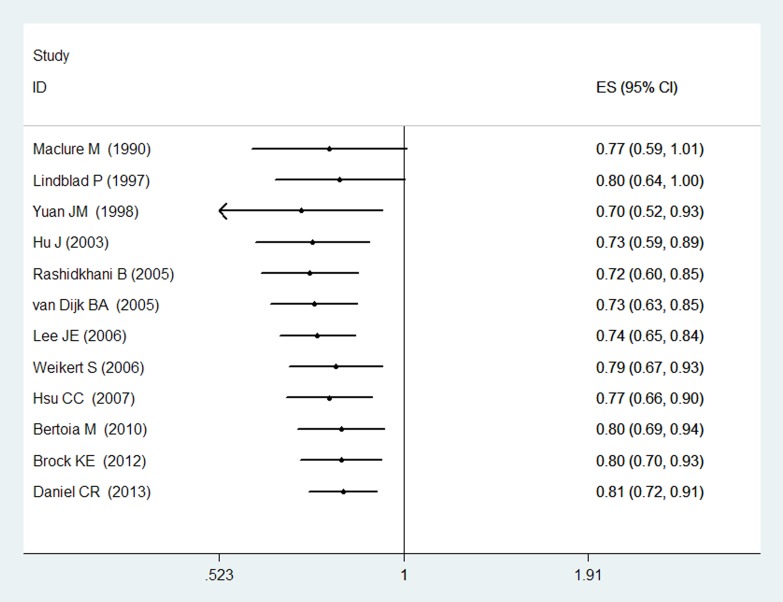
Forest plot: cumulative meta-analysis of CV intake and RCC risk.

The association of CV intake with RCC risk in each subgroup are shown in Table 4. The protective effect of CV intake was still significant when analyses were restricted to 6 high-quality studies (RR = 0.89, 95% CI [0.82, 0.98]). Statistically significant protective effects of CV intake on RCC were observed both among cohort studies (RR = 0.92, 95% CI [0.84, 1.00]) and case-control studies(RR = 0.72, 95% CI [0.64, 0.81]). When stratified the various studies by population, CV intake was significantly associated with reduced RCC risk among studies conducted in America (RR = 0.77, 95%CI [0.70, 0.86]), however, CV intake had no significantly association with RCC risk among studies conducted in Europe (RR = 0.87, 95%CI [0.71, 1.07]). No significantly association was observed in both male(RR = 0.99, 95%CI [0.86, 1.15]) and female population(RR = 0.80, 95%CI [0.59, 1.07]). When we examined whether the associations differed by adjustment for smoking status, alcohol intake, meat intake, total energy intake, or hypertension status, the associations did not vary by these factors. However, the number of adjustment factors affected the combined RR greatly, the inverse association between CV intake and RCC risk appeared to be weaker with a smaller number of adjustment factors(n≤7), and the difference was not statistically significant (RR = 0.87, 95%CI [0.74, 1.01]) (Table 4). To test the robustness of association, sensitivity analyses were carried out by excluding studies one-by-one. Sensitivity analysis indicated that no significant variation in combined RR by excluding any of the study, confirming the stability of present results. A cumulative meta-analysis of total 12 studies was carried out to evaluate the cumulative effect estimate over time. In 1990, Maclure M et al [21] reported a effect estimate of 0.77 (95% CI [0.59, 1.01]). Between 1990 and 2005, five studies were published, with a cumulative RR being 0.73 (95% CI [0.63, 0.85]). Between 2005 and 2013, six more publications were added cumulatively, resulting in an overall effect estimate of 0.81 (95% CI [0.72, 0.91])(Fig. 3).

**Table 4:** Summary risk estimates of the association between cruciferous vegetable consumption and renal cell carcinoma risk.

	No. of studies	Pooled estimate		Tests of heterogeneity	
		RR	95% CI	P value	I^2^(%)
All studies	12	0.81	0.72–0.91	0.01	55.7
High-quality studies (scores≥7)	6	0.89	0.82–0.98	0.37	7.2
Study design					
Cohort	6	0.92	0.84–1.00	0.16	37.4
Case–control	6	0.72	0.64–0.81	0.30	18.0
Population based	5	0.73	0.62–0.87	0.22	30.1
Hospital based	1	0.68	0.55–0.84	-	-
Geographic location					
Europe	6	0.87	0.71–1.07	0.02	63.2
America	6	0.77	0.70–0.86	0.23	27.1
Gender					
Male	3	0.99	0.86–1.15	0.32	13.8
Female	4	0.80	0.59–1.07	0.05	61.0
Adjusted for confounders					
Number of adjustment factors					
n≥8 confounders	7	0.78	0.66–0.91	<0.01	68.3
n≤7 confounders	5	0.87	0.74–1.01	0.23	29.3
Major confounders adjusted					
Alcohol					
yes	7	0.86	0.75–0.98	0.03	56.6
no	5	0.70	0.59–0.82	0.25	26.0
Smoking status					
yes	10	0.82	0.72–0.94	0.01	61.4
no	2	0.74	0.58–0.94	0.55	0.0
Meat intake					
yes	2	0.76	0.63–0.92	0.13	56.1
no	10	0.82	0.71–0.96	0.02	56.0
Hypertension					
yes	7	0.80	0.67–0.96	0.04	53.9
no	5	0.81	0.67–0.98	0.03	63.3
Total energy intake					
yes	5	0.89	0.82–0.97	0.33	13.8
no	7	0.76	0.62–0.93	0.03	58.1

RR = Relative risk; CI = confidence interval.

### Meta-regression analysis

To better investigate the possible sources of between-study heterogeneity, a meta-regression analysis was performed. Study design (cohort, case-control), geographic area(Europe, America), publication year, major confounders adjusted(smoking status, alcohol intake, meat intake, total energy intake, hypertension status), which may be potential sources of heterogeneity, were tested by a meta-regression method. Only study design had statistical significance in a multivariate model ( P = 0.035).

### Publication bias

**Figure 4:**
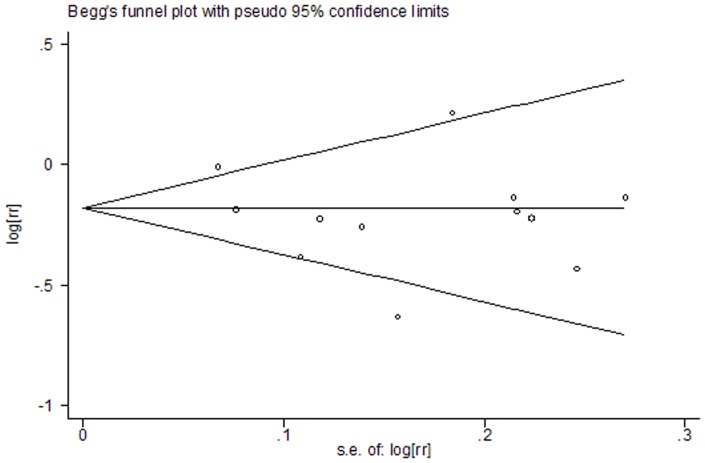
Funnel plot for publication bias in the studies investigating risk for RCC associated with CV intake.

In the present meta-analysis, no publication bias was observed among studies using Begg's P value ( P = 0.21) and Egger's (P = 0.35) test, which suggested there was no evidence of publication bias (Fig. 4).

## Discussions

This is the first meta-analysis evaluating the association between CV intake and RCC risk. Six cohort and six case-control studies were included in the present analysis, involving 1,228,518 participants and 5,773 RCC cases. Finally, we found that intake of CV may reduce the risk of RCC in humans (comparing the highest with the lowest category). In the present meta-analysis, significant heterogeneity was observed among all studies. Therefore, a random-effects model, which provides a more conservative standard error and a larger confidence interval, was chosen over a fixed-effects model to determine the pooled RR estimates in our meta-analysis. Meta-regression analysis suggested that study design was the sources of between-study heterogeneity. When we did subgroup analysis by study design, the heterogeneity decreased greatly, which further suggested study design was the sources of between-study heterogeneity.

In our subgroup analyses, the results were not substantially affected by study design. Cohort and case–control studies alone showed inverse association between CV intake and RCC risk. However, we found a significant risk reduction in RCC in American populations but not in European populations. The reason for the difference is unclear. The differences in genetic susceptibility, culture, and lifestyles may explain part of the inconsistency of the results. Another possible reason is the difference in ethnic composition between European and American population. No significantly association was observed in both male and female population, however, we should notice that only 4 studies studying the association between CV intake and RCC risk. That number is rather low to draw firm conclusions. Most of the included studies didn't reported results separately for males and females. So, future studies should reported results separately for males and females. As we know, RCC is more incident in males than females, and the effect of CV intake may be different between males and females. When we examined whether the associations differed by adjustment for smoking status, alcohol intake, meat intake, total energy intake, or hypertension status, we did not find any substantial differences, indicating that the influence of those adjusted confounders on the results might be small. However, the number of adjustment factors affected the combined RR greatly, the inverse association between CV intake and RCC risk appeared to be weaker with a smaller number of adjustment factors(n≤7), and the difference was not statistically significant. Maybe different adjustment factors combine to affect the results significantly, however, the influence of individual factors is small. Cumulative meta-analyses show that the estimates gradually became consistent, and the corresponding CIs narrowed down with the increase of the number of included studies in the order of publication year. Sensitivity analysis indicated that an omission of any studies did not alter the magnitude of observed effect, suggesting a stability of our findings. Moreover, the results of Begg's test and Egger's test did not support the existence of major publication bias.

The inverse association between CV intake and risk of RCC is biologically plausible.

Previous meta-analyses have suggested that CV intake may reduce the risk of colorectal cancer [38], gastric cancer [39], female lung cancer [40], bladder cancer [41], and prostate cancer [42]. Our finding was in accordance with the findings of the above meta-analyses.

The strength of the present analysis lies in inclusion of 12 studies, reporting data of 1,228,518 participants and 5,773 RCC cases. Publication bias, which, due to the tendency of not publishing small studies with null results, was not found in our meta-analysis. Most studies adjusted for some potential confounders, including smoking status, BMI, hypertension, alcohol consumption, and total energy intake. Furthermore, our findings were stable and robust in sensitivity analyses. There were several limitations in our meta-analysis. First, we did not search for unpublished studies, so only published studies were included in our meta-analysis. Therefore, publication bias may have occurred although no publication bias was indicated from both visualization of the funnel plot and Egger's test. Second, as we know, CV includes a group of vegetables such as broccoli, cauliflower, cabbage and brussels sprouts and other members of the family. However, we only assessed total CVs consumption and RCC risk. We haven't done subgroup analysis of different types of CVs, for a lack of data. Third, none of the included studies separate the CV intake by cooking factors. Cooking, particularly boiling and microwaving at high power, may decrease the bioavailability of ITCs [12]. Fourth, as the observational nature of the data, it is possible that the observed significant inverse association between CV intakes and risk of RCC could be due to unmeasured or residual confounding. Fifth , we haven't done sub-group analysis by ethnicity. Last but not least, due to different methods used to report CV consumpion among studies, we failed to carry out a dose – response analysis between CV consumption and RCC risk.

In conclusion, the findings of this meta-analysis, suggested that high intake of CV was associated with the reduced risk of RCC among Americans. More studies, especially high quality cohort studies with larger sample size, well controlled confounding factors are warranted to confirm this association. More in-depth studies are warranted to report more detailed results, including other specific vegetables within the CV family, stratified results by gender, cooking methods, or adjustment for potential confounders, such as physical activity, healthy diet.

## Supporting Information

Checklist S1 **PRISMA Checklist.**
(DOC)
